# Exploratory study examining the at-home feasibility of a wearable tool for social-affective learning in children with autism

**DOI:** 10.1038/s41746-018-0035-3

**Published:** 2018-08-02

**Authors:** Jena Daniels, Jessey N. Schwartz, Catalin Voss, Nick Haber, Azar Fazel, Aaron Kline, Peter Washington, Carl Feinstein, Terry Winograd, Dennis P. Wall

**Affiliations:** 1 Division of Systems Medicine, Department of Pediatrics, Stanford University, Palo Alto, CA USA; 2 Department of Computer Science, Stanford University, Palo Alto, CA USA; 3 Department of Psychiatry and Behavioral Sciences, Stanford University, Palo Alto, CA USA; 4 Department of Biomedical Data Science, Stanford University, Palo Alto, CA USA

**Keywords:** Translational research, Social behaviour, Empathy, Autism spectrum disorders

## Abstract

Although standard behavioral interventions for autism spectrum disorder (ASD) are effective therapies for social deficits, they face criticism for being time-intensive and overdependent on specialists. Earlier starting age of therapy is a strong predictor of later success, but waitlists for therapies can be 18 months long. To address these complications, we developed Superpower Glass, a machine-learning-assisted software system that runs on Google Glass and an Android smartphone, designed for use during social interactions. This pilot exploratory study examines our prototype tool’s potential for social-affective learning for children with autism. We sent our tool home with 14 families and assessed changes from intake to conclusion through the Social Responsiveness Scale (SRS-2), a facial affect recognition task (EGG), and qualitative parent reports. A repeated-measures one-way ANOVA demonstrated a decrease in SRS-2 total scores by an average 7.14 points (*F*(1,13) = 33.20, *p* = <.001, higher scores indicate higher ASD severity). EGG scores also increased by an average 9.55 correct responses (*F*(1,10) = 11.89, *p* = <.01). Parents reported increased eye contact and greater social acuity. This feasibility study supports using mobile technologies for potential therapeutic purposes.

## Introduction

Children with autism spectrum disorder (ASD) struggle to recognize facial expressions, make eye contact, and engage in social interactions.^1,2^ An estimated 1 in 68 children have an ASD, and many can have dramatic improvements if social skills are taught intensively from an early age.^1–5^ Children with ASD have demonstrated deficits in facial processing abilities, such as distinguishing fear from surprise and identifying subtler emotions.^6–9^ Children also struggle with facial engagement and eye contact.^10,11^ Teaching these skills to children with autism is important for social development and is closely linked with empathy.^12–16^

Today’s standard for treatment of these core ASD deficits focuses on a form of behavioral therapy known as applied behavioral analysis (ABA).^17,18^ Although ABA therapy is effective in increasing IQ, improving eye contact, face-to-face gaze, and emotion recognition, children who receive ABA often struggle to generalize learned behaviors to natural interactions and are dependent on prompts.^17,19^ Therapies called naturalistic developmental behavioral interventions (NDBIs) promote better generalization of newly learned skills due to their integration into the child’s natural everyday interactions.^15,20,21^ However, the delivery of behavioral interventions like ABA and NDBIs is bottlenecked by an increasing imbalance between the availability of behavioral therapists and the number of children who must receive care.^22,23^

One of the strongest predictors of greater treatment outcomes is younger age at entry into behavioral interventions^24,25^, but delays in access to therapy leave many children untreated until after sensitive periods for language and cognitive development have already passed.^26,27^ Additionally, waitlists for access to therapies, such as ABA and NDBIs can be up to 18 months long.^23,25^ Consequently, many children with autism are unable to build such core social skills and subsequently regress towards a path of isolation.^24,28^ These issues have compounded into an urgent need for alternative, ubiquitous mobile methods of delivery^29^ that can positively alter the healthcare system and scale to meet the growing population in need of early intervention.

To address the complications associated with accessing the clinical setting and to expedite children’s access to therapy, we have begun development of a system to deliver therapy at home using a machine-learning-assisted software system that runs on Google Glass paired with an Android smartphone, designed for use in the child’s natural environment during social interactions with friends and family members.^30–32^ It recognizes eight emotions, as described in detail by Ekman et al.: happiness, sadness, anger, disgust, surprise, fear, neutral, and contempt (named “meh” in child-friendly terms), which are recognized as theoretically universal emotions.^33–35^ The Glass provides audiovisual feedback to the wearer that corresponds to which of the eight emotions the Glass recognizes during social interactions through its outward-facing camera.

Using technology and software for children with autism may assist children struggling with social anxiety in social interactions.^36–38^ In addition, incorporating visual, dynamic, and real-world stimuli can increase learning and greater generalizability to other in vivo interactions.^36,39^ Studies such as those conducted by Madsen et al.^40^ and Liu et al.^41^, among others^42^, have utilized mobile technologies like portable PCs and Google Glasses to assist children with autism during social interactions via software for facial recognition, eye tracking, and structured games. While more recent projects incorporate social interactions with the use of technology, an important improvement on the use of computer programs, the limitations of most of these technology-based intervention studies include potentially distracting software,^40–42^ a limited range of participants’ autism severities,^41^ adolescent participant populations,^40^ and highly structured games (rather than naturalistic interactions).^41,42^ We seek to improve upon these approaches with a wearable system that can seamlessly augment social interactions with social learning cues in an unobtrusive, naturalistic way. We hypothesize that our system’s ability to provide continuous behavioral therapy outside of clinical settings will enable faster gains in social acuity, and that within a limited and self-directed period of use, will permit the child to engage in increasingly more complex social scenarios on his/her own.

Our pilot research^9,30,43^ established smart glasses as a practical and feasible platform to deliver audio–visual feedback to children with ASD. Part of the foundation of this pilot work included an iterative design aspect, where, throughout the study, we evaluated and compared various interfaces and games. The human–computer interaction lessons from our iterative design process have been documented in Washington et al.^43^ We created robust facial expression software^31,45,46^ and implemented many design-focused iterations.^32,47^ The present study expands upon our previous work and sends our prototype system to the home environments of children with ASD. In this study, we evaluated the potential of the Superpower Glass prototype as a wearable therapy intervention that increases social skills, facial affect recognition, and eye contact for children with autism between the ages of 3 and 17 years. Additionally, this field study was designed to determine feasibility of the fit and form factor of the Glasses, the appropriate “dosage” as determined by family usage over several weeks, and the feasibility of sustained use of Superpower Glass in the home setting.

## Results

Between July 2016 and October 2016, 24 participants consented to participate and attended an intake appointment at Stanford University. Five families withdrew prior to using the Superpower Glass system at home or were unable to continue the treatment portion of the study due to conflicts in personal schedules. All five of these participants who withdrew prior to using the Superpower Glass system at home were male, with an average age of 7 years 6 months (SD = 2.51 years), and had an average ABIQ score of 95.8 (median = 106, SD = 27.95). Four of these participants were Caucasian and one was Asian. The research team excluded five additional families who did not comply with the at-home study procedures, which required families to complete three or more sessions with Superpower Glass per week, for 20 min per session. The five families that were excluded by the research team due to noncompliance with the required use of the device were also all male, with an average age of 8 years 6 months (SD = 4.04 years), and had an average ABIQ score of 74.8 (median = 76, SD = 10.08). Two of these participants were Caucasian and three were Asian. This yields a study compliance rate of 73.68%. The following results are based on the remaining 14 families. Refer to Table 1 for participant demographics.

**Table 1:** Participant demographic information

Demographics *n* = 14	Sub-category	Mean (SD)/percent (*n*)
Age (years)		9.57 (3.37)
Gender (% male)		79.58% (*n* = 11)
Diagnosis	ASD diagnosis (DSM-5)	79.58% (*n* = 11)
	Asperger’s diagnosis (DSM-IV)	21.42% (*n* = 3)
	Confirmed diagnosis (via ADOS report)	100% (*n* = 14)
Comorbidity	Comorbid diagnoses of ASD/PDD-NOS, ADHD, Generalized anxiety disorder, clinical depression, and a learning disability	7.14% (*n* = 1)
	Comorbid diagnoses of ASD, ADHD, and dysgraphia	7.14% (*n* = 1)
	Comorbid diagnoses of ASD and ADHD	14.28% (*n* = 2)
	Only diagnosed with an ASD	72.43% (*n* = 10)
Race	Caucasian/White (%)	42.85% (*n* = 6)
	Asian (%)	50.00% (*n* = 7)
	Native Hawaiian or other Pacific Islander (%)	7.14% (*n* = 1)
Ethnicity	Hispanic/Latino/Spanish origin (%)	7.14% (*n* = 1)
	Non-hispanic/latino (%)	92.86% (*n* = 13)
*Clinical evaluations*		
Social Communication Questionnaire (SCQ^73^)		21.64 (6.79)
Stanford Binet ABIQ^76^, total standard score		95.14 (22.36)
ABIQs for each ASD severity classification	Low-functioning ASD severity (*N* = 4)	73.80 (15.95)
	Moderate ASD severity (*N* = 5)	92.20 (7.53)
	High-functioning ASD severity (*N* = 5)	118.00 (9.72)
Child behavior checklist (CBCL^72^)	Total problems	65.21 (7.88)
	Internalizing problems	64.57 (9.30)
	Externalizing problems	58.43 (6.02)
	Stress problems	64.86 (8.14)
	Depressive problems	63.93 (9.19)
	Anxiety problems	65.21 (11.76)
	Attention deficit/hyperactivity problems	61.93 (10.02)
	Oppositional defiant problems	59.21 (7.28)
Multidimensional Social Competence Scale (MSCS^77^)	Total score	198.31 (31.13)
	Social motivation	26.23 (7.87)
	Social inference	25.15 (7.57)
	Demonstrating empathic concern	28.00 (7.48)
	Social knowledge	28.31 (7.15)
	Verbal conversation skills	27.38 (7.76)
	Nonverbal conversation skills	33.54 (6.79)
	Emotional regulation	29.69 (3.95)

The 14 families who complied with the minimum usage requirements had the device at home for an average of 72 days, or 10.29 weeks (SD = 5.0 weeks, max = 19.43 weeks, min = 3.86 weeks). In total, we gathered 5726 min of app usage logged data. Participant mean app usage was 409 min (SD = 266.8 min, max = 1010 min). Of the 14 families, three families used the device for 1 month (SD = 1.53 days) for an average of 10.27 sessions per week (SD = 2.18), two families used the device for more than 1 month but less than 2 months (SD = 12.73 days) for an average of 9.14 sessions per week (SD = 7.74), five families used the device for more than 2 months but less than 3 months (SD = 8.38 days) for an average of 4.39 sessions per week (SD = 2.50), three families used the device for more than 3 months but less than 4 months (SD = 13.08 days) for an average of 3.29 sessions per week (SD = .71), and one family used it for 4.5 months for an average of 3.76 sessions per week.

We report the clinical implications of the feedback families gave during the semi-structured interview. Detailed design and user experience feedback is reported in a previous publication.^43^ In summary, families found the system to be engaging, useful, and fun based on feedback from their conclusion interviews (Table 2; Notable responses are recorded in Appendix A). According to the Superpower Glass logged data, families chose evenly between structured interactive games (Capture the Smile and Guess the Emotion) and Free Play (51% to 49%). However, activity preference varied substantially between participant ASD severities based on both logged data and qualitative feedback. Families whose children had more social-cognitive deficits preferred the structured game modes and chose to run far fewer Free Play sessions.^43^ Linear regression analysis of game mode selection compared to ABIQ showed significant correlations as ABIQ decreased, Free Play selection decreased and Guess the Emotion selection increased (*p* = 0.026 and *p* = 0.040, respectively).

**Table 2:** Interview questions asked during conclusion appointments

Interview questions to parents	Responded “yes”
(1) Do you feel that additional games and/or more complex games would increase your engagement?	*N* = 14 (100%)
(2) Did you find charging the device to be burdensome or challenging?	*N* = 14 (100%)
(3) Did you use some sort of a reward system to get your child to use the system?	*N* = 5 (35.7%)
(4) Would you use the system more if the entire experience was gamified?	*N* = 14 (100%)
(5) During the sessions, did you ever change your facial expression to be more emotive as a result of the emotion recognition accuracy?	*N* = 6 (42.9%)
(6) Did you find that your child made increased eye contact when not wearing the device?	*N* = 12 (85.7%)
(7) Did you find that your child made increased social interaction when not wearing the device?	*N* = 7 (50%)
(8) Did you find that your child exhibited increased emotional recognition when not wearing the device?	*N* = 11 (78.6%)
(9) Did you find that your child increased spontaneous conversation when not wearing the device?	*N* = 2 (14.3%)
(10) Did you find that your child showed increased patience when not wearing the device?	*N* = 4 (28.6%)
(11) Did you find that your child showed increased empathy when not wearing the device?	*N* = 6 (42.9%)
(12) Did you find that using the device resulted in an overall increase in quality family time?	*N* = 13 (92.9%)
*Interview questions to children*	
(1) Would you want to use this tool in social settings outside of the home?	*N* = 12 (85.7%)
(2) (Asked only to 10 participants who had siblings) Did you use the device with your siblings?	*N* = 5 (50%)
(3) Did you ever try to disable to Superpower Glass application on the Android device to access the other features of the phone?	*N* = 7 (50%)
(4) Did you find the technical component of the system “cool”?	*N* = 10 (71.4%)
(5) Did the Google Glass make you interested in the study?	*N* = 10 (71.4%)
(6) Did you enjoy playing the games provided with the system?	*N* = 14 (100%)
(7) Do you feel that additional games and/or more complex games would increase your engagement?	*N* = 14 (100%)
(8) Did you notice the Google Glass heating up when using it?	*N* = 10 (71.4%)
(9) Did you notice errors in the software’s emotion recognition capabilities?	*N* = 10 (71.4%)
(10) Would you use the system more if the entire experience was gamified?	*N* = 14 (100%)
(11) Would you prefer a more personalized experience?	*N* = 10 (71.4%)
(12) Did you enjoy the free play activity?	*N* = 11 (78.6%)
(13) Did you enjoy the guess the emotion activity?	*N* = 13 (92.9%)
(14) Did you enjoy the capture the smile activity?	*N* = 11 (78.6%)
(15) What was your preferred method of feedback: both audio and visual?	*N* = 12 (85.7%)
What was your preferred method of feedback: visual feedback only?	*N* = 2 (14.3%)
What was your preferred method of feedback: audio feedback only?	*N* = 0 (0%)

Specific to the clinical implication of Superpower Glass, 12 of 14 families commented that they had observed an increase in eye contact from intake to conclusion during the semi-structured interview.

The mean total SRS-2 score during the intake appointments was 80.07 (SD = 9.53, SEM = 2.55); the mean total SRS-2 score during the conclusion appointments was 72.93 (SD = 10.29, SEM = 2.75). Children’s total SRS-2 scores decreased an average of 7.38 points over the course of the study (*F*(1,13) = 33.20, *p* < .001, a higher score indicates a higher severity of ASD) and there was no significant correlation between the decrease in SRS-2 scores to the number of days the device was at home or to the ASD severity category of ABIQ (Table 1). Six participants moved from one SRS-2 severity class of autism to a less severe class (four from “severe” to “moderate”, one from “moderate” to “mild”, and one from “mild” to “normal”). A repeated-measures one-way ANOVA analysis showed a significant decrease in total SRS-2 score from start to end of the prescribed Glass usage, and for each subsection of the SRS-2 (Table 3). None of the participant’s scores increased between measurements. There was no significant correlation between ABIQ, number of therapies enrolled, nor days of Superpower Glass at home, and points of improvement on the SRS-2 Total *T*-score (respectively, *p* = .108, *p* *=* .247, and *p* = .374).

**Table 3:** Statistics from a repeated measures ANOVA analysis of the SRS-2 at intake and at conclusion

SRS-2 subsection T-scores	Mean (SD)		Sig.	SEM	
	Intake	Conclusion		Intake	Conclusion
SRS total	80.07 (9.531)	72.93 (10.292)	*p* < .001	2.547	2.751
SRS social awareness	78.07 (11.194)	71.21 (11.544)	*p* < .001	2.992	3.085
SRS social cognition	74.86 (8.160)	69.93 (10.594)	*p* < .05	2.181	2.831
SRS communication	78.93 (10.477)	72.57 (10.308)	*p* < .001	2.8000	2.755
SRS social motivation	68.71 (9.523)	64.79 (9.784)	*p* < .05	2.545	2.615
SRS autistic mannerisms	83.07 (18.036)	72.07 (11.737)	*p* < .01	4.820	3.137

In addition, the SRS-2 data showed significant changes pre-Glass and post-Glass usage on sub-domain questions, including changes in recognizing intent, social initiation, social interaction, and eye contact. The Wilcoxon Rank Sum test run on the SRS-2 65 item-level questions showed a nominally significant change from intake to conclusion for five items among the 65 (Table 4).

**Table 4:** SRS-2 Item-level analysis based on a Wilcoxon Rank Sum test and Benjamini–Hochberg correction

SRS-2 question	Domain	Mean (SD)		Mean change	Uncorrected sig.	Sig. after correction (two-tailed)
		Intake	Conclusion			
Q5. Doesn’t recognize when others are trying to take advantage of him or her.	Theory of mind	3.29 (.73)	2.64 (.84)	−.65 (less true)	.003	.033
Q23. Does not join group activities unless told to do so.	Social initiation	2.86 (.95)	2.36 (.93)	−.50 (less true)	.020	.055
Q30. Becomes upset in a situation with lots of things going on.	Sensory sensitivity	3.00 (.78)	2.36 (.84)	−.74 (less true)	.021	.055
Q35. Has trouble keeping up with the flow of a normal conversation.	Social overtures	2.93 (.73)	2.14 (.95)	−.79 (less true)	.005	.033
Q45. Focuses his or her attention to where others are looking or listening.	Eye contact/joint attention	1.79 (.42)	2.36 (.84)	+.57 (more true)	.021	.055

Due to our iterative design platform for this pilot work, the first three study participants did not receive the EGG evaluation at intake. The remaining 11 of the 14 participants completed the EGG at intake and conclusion. The 11 participants’ EGG scores yielded a significant increase in emotion labeling accuracy (*F*(1,10) = 11.893, *p* = .006) (Table 5). There was no significant correlation between ABIQ, number of therapies enrolled, nor days of Superpower Glass at home, and points of improvement on the EGG (*p* = .499, *p* = .793, and *p* = .271, respectively).

**Table 5:** ANOVA analysis on the Emotion Guessing Game from intake to conclusion

Emotion Guessing Game, sample size	Mean (SD)			*p*-value
	Intake	Conclusion	*F*	
*N* = 11	28.45 (11.00)	38.00 (2.68)	11.893	.006

## Discussion

Significant decreases in SRS-2 total scores and subscores, concomitant increases in emotion recognition measured by EGG, and responses to semi-structured interviews support the hypothesis that the use of Superpower Glass may be an effective and practical wearable therapy intervention for children with autism that can increase social skills, facial affect recognition, and eye contact. Since neither the number of days with Glass at home nor the child’s ABIQ score were significantly correlated with improvements on both outcome measures from Pearson’s correlation tests, this initial finding may suggest that the system was equally effective for all children in our study, irrespective of length of time with Superpower Glass at home (between 4 and 19 weeks), and ABIQ score. However, the significant change demonstrated by participants from the SRS-2 must be treated with caution, as we did not include a comparison control group for comparison.

Twelve of the 14 families commented during the semi-structured interview that they observed an increase in eye contact from intake to conclusion. This is also supported by one of the significant SRS-2 question items focused on eye contact, which was included in the Wilcoxon Rank Sum test analysis for changes from intake to conclusion. The implications of this finding suggest that Superpower Glass may improve eye contact among children with autism, although these findings were not compared to a control group and thus none of the results are conclusive. This will be examined in future studies using a control group and using more quantitative approaches.

### Limitations

While this exploratory study provided useful insights to implementation of the study tool at home and the potential of a wearable behavioral therapy system for children with ASD, there were limitations to the study’s ability to prove clinical efficacy of our learning aid. Families were required to attend at least three in-person appointments at Stanford University. Stanford University is situated in an area that is highly enriched for familiarity with wearables and technology, and as families were required to attend at least three in-person appointments at Stanford, our sample population may have resulted in a high concentration of tech-savvy families and children who participated. Our results should be treated cautiously, as families without technical backgrounds may find their experience with our system less intuitive, and thus, further validation of Superpower Glass is necessary to understand efficacy for the diverse autism population.

Our primary outcome measure, the SRS-2, is parent-reported. We did not otherwise collect any experimental or measurement strategies on the participant’s social skills gains to determine if the statistically significant SRS-2 score reduction was due to a parent placebo effect.^48^ In our upcoming RCT, as described in Future Work, we will ameliorate this by including child-directed evaluations conducted by a blinded researcher (such as the Brief Observation of Social Communication Change^49^ and NEPSY-II: Affect Recognition^50^).

The Superpower Glass software was updated several times throughout the study. Updates to the software included improvements to camera and network performance, video recording stability, device connectivity, and visual and audio feedback response; infrastructural changes for gathering consent and transferring data; and user experience adjustments to the presentation of the various options available for therapy sessions. Though participants started and ended the study with different versions of Superpower Glass software, there were no significant functional changes to the software or instructions to complete the study procedures that would differentiate their intervention experience.

The significant changes on the evaluations found in this study were not compared to a control group, and therefore we cannot determine whether it was the system itself, a maturation effect, or another aspect of the intervention process that resulted in the changes observed. A follow-on study with a larger, randomized participant population can determine these effects and confirm our hypothesis that our Superpower Glass system can lead to sustained gains in social acuity. Furthermore, while all our other collected evaluations were peer-reviewed and standardized, EGG was a novel, unstandardized assessment. There is potential that a learning effect contributed to the increase in EGG scores for each participant. Additionally, the same administrator conducted all EGG assessments and because the EGG emotions are expressed to the child during an in-person assessment, there was potential for human error. Finally, baseline EGG scores for the first three families were not collected until midway through their participation. This introduces different sample sizes in our two outcome measures. However, we still see significant decreases in our other primary outcome measure, the SRS-2, when we exclude the same first three participants from EGG analysis. The same repeated measures ANOVA analysis on the SRS-2 with these *N* = 11 participants continues to demonstrate significance of *p* < .05 in all SRS subscores from intake to conclusion, except for the Social Cognition (*p* = .071) and Social Motivation (*p* = .300) subscales.

Lastly, limitations from the hardware included a short battery life and difficulties with charging. Dealing with these limitations inherently limited our participants’ study tool usage; however, our software has been coded in such a way that it can be ported to other wearable platforms, should a similar kind become available, therefore ameliorating the above hardware limitations for potential future studies.

### Future work

Further testing and refining needs to be done to validate our Superpower Glass system as a home behavioral therapy tool. The results from this exploratory and feasibility study have provided us with a valuable preliminary understanding of the way that parents and children with autism engage with our Superpower Glass system, an important step before beginning a larger randomized control trial to determine efficacy.^51^ As such, this study has paved the way towards creating a more robust protocol via a blinded, crossover randomized control trial with a cohort of at least 50 families, including additional and standardized outcome measures to closely assess changes in emotion recognition, eye contact, and social skills that our Glass system may encourage in children with autism. We intend to benchmark gains in social acuity over the course of 12 weeks at intake, conclusion, and follow-up to the Superpower Glass therapy program and run an intention-to-treat analysis.

## Methods

### Participant recruitment and screening methods

Under a Stanford University approved Institutional Review Board protocol, we identified eligible participants with autism spectrum disorder (ASD) by referral from the Autism and Developmental Disabilities Clinic and the Developmental Behavioral Unit of Lucile Packard Children’s Hospital. Referrals were also generated by conference presentations from Stanford faculty and staff, educational presentations at ABA therapy organizations, social media outreach, and press outreach.^52–55^

Informed consent was obtained from all participants in accordance with our approved IRB, participants then completed a REDcap^56^ survey and phone interview to screen for eligibility. The screening questionnaire asked families to provide general contact, demographic, and diagnostic information (Table 1). We excluded families if their child: (1) had evidence of a genetic, metabolic, or infectious etiology based on medical history; (2) had a history of seizures or other neurological problems; and/or (3) had a diagnosis of severe mental disorders such as schizophrenia or bipolar disorder. Eligible participants were asked to complete a phone screen during which the study manager conducted the Social Communication Questionnaire (SCQ^57^). Families were included in the study if: (1) their child scored > 15 on the SCQ; (2) the child had a medical diagnosis of ASD and provided the study manager with the diagnostic report, ascertained by an Autism Diagnostic Observation Schedule (ADOS)^58^ conducted by a clinician and based on the Diagnostic and Statistical Manual of Mental Disorders (DSM-IV or DSM-5) criteria;^59,60^ and (3) the child was between 3 and 17 years old.

### Study tool

**Fig. 1:**
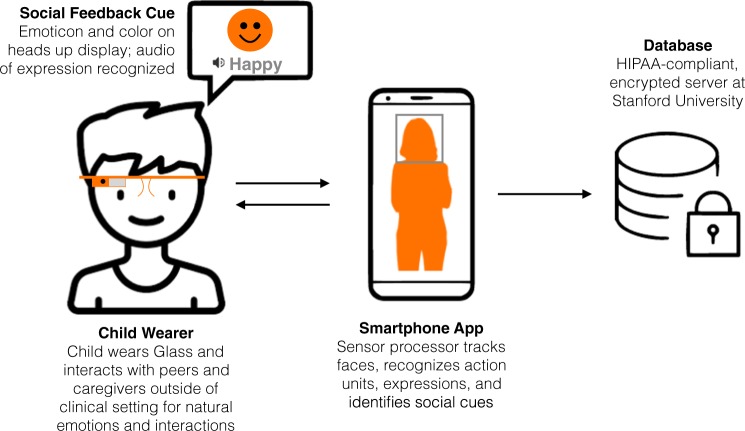
Overview of the Superpower Glass System. The Android app connects to the Google Glass worn by the child participant. The app receives facial-tracked data from the Glass camera and computes the emotion expressed by the person the child is interacting with and returns the emotion as social feedback to the child, all the while recording the social interaction for parent–child review when the session is complete

The Superpower Glass system is a machine-learning-assisted therapeutic software system ^32,45–47^ that combines Google Glass (worn by the child) with a wirelessly linked Android phone application (“app”), designed to teach children with autism how to interpret eight universal emotions in faces^33–35^ (Happy, Sad, Angry, Scared, Surprised, Calm, Disgust, and “Meh”) to improve overall social awareness and increase eye contact during social interactions in the child’s natural environment. Figure 1 provides an overview of the system’s architecture.

**Fig. 2:**
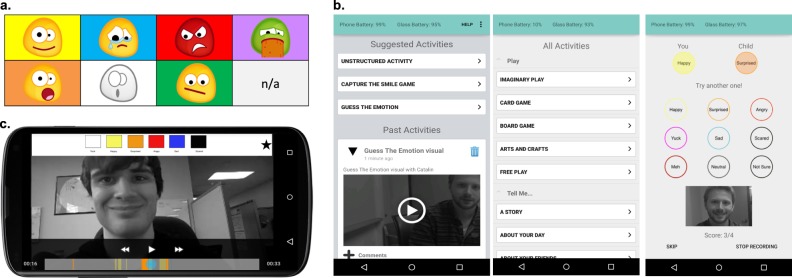
Example of the superpower glass–system interface. **(a)** Emoticons are displayed on the Google Glass heads-up display, corresponding to the emotion recognized by the emotion classifier in real-time. **(b)** The app dashboard on the Android phone allows parents to scroll through a newsfeed of previously recorded sessions or to start a new session. Parents can select the game mode on the app dashboard. Selecting “Unstructured Activity” produces a list of activities for Free Play (the selection does not change the experience). Selecting “Guess the Emotion” produces the screen on the right. **(c)** Parents and children can review a recorded video session on the Android phone app with a color-coded scrub bar representing when each emotion was expressed and recognized by the system during the video session

During this study, participants were provided game mode options and feedback selection options at the beginning of each session via the app. Each game mode is focused on an aspect of emotion recognition and social interaction, such as emotion identification, eye contact, and/or social initiation.^10^ Before the start of a Superpower Glass session (a period of time when the child and their family member(s) interact with the system’s modes), the parent selects the game mode on the app dashboard, and then selects the type of feedback that will be provided to the child via Google Glass. The feedback options are audio feedback labeling the recognized emotion via the bone-conducting speakers on the Google Glass, emoticons representing the recognized emotion via the heads-up display on the Google Glass, or a combination of both audio and emoticons. When a session is started, the Glass’s outward-facing external camera captures video data of the child’s field of view, which is then passed to the app and saved at a rate of 30 frames per second. When a face is in the camera’s field of view expressing one of the eight emotions, the app automatically classifies the emotion and provides real-time feedback to the child-wearer, via Google Glass, using the mode of feedback selected at the start of the session. The social cues are displayed in the Glass’s peripheral monitor (as audible words, emoticons, or both, depending on the feedback selection) and the audio cues use Glass’s bone-conduction speaker. At the end of a session, the parent and child can review the recorded social interaction videos in the app’s dashboard, enabling a dialog about social interactions. The recorded videos’ scrub bar is color-coded, corresponding to when the classifier recognized emotions. Figure 2 demonstrates (a) the feedback emoticons, (b) the game modes, and (c) the parent review feature within the app.

### Emotion expression recognition software

The emotion classification system was constructed by logistic regression classification that evaluates extracted HOG features^61^ of a face (captured by Google Glass’ outward facing camera), accounts for various lighting environments,^62^ and uses neutral subtraction^31^ to better discriminate between the eight expressions (Happy, Sad, Angry, Scared, Surprised, Calm, Disgust, and “Meh”). The base emotion classification model was trained on a mix of publicly available databases of labeled facial expressions (Binghamton University 4D Facial Expression Database; ^63^ the Bosphorus Database;^64^ the CMU Pose, Illumination, and Expression Database;^65^ the Extended Cohn–Kanade Dataset;^66^ the Japanese Female Facial Expression Database;^67^ the MMI Facial Expression Database;^68^ and MultiPIE;^69^ along with our own data gathered in-lab via another approved protocol through Stanford University Institutional Review Board). Data were then augmented by mirroring images and adding Gaussian field noise to the face tracker to simulate tracking inaccuracy. To construct user-specific models from the base model, we then employed hierarchical Bayesian domain adaptation,^70^ which put a Gaussian prior on the distribution of model coefficients. User-specific models were created during participants’ first study appointment and the process took roughly five minutes per family member. This in-house pipeline of the emotion classifier that runs on the Superpower Glass system was engineered for real-time performance in diverse home settings, with special considerations for conditions, such as specific family members, lighting, and pose variance. Washington et al.^43,47^ further details the evaluation of and model accuracy for the adapted emotion classification model.

### Game modes

#### Free Play: An unstructured activity developed to increase eye contact, enhance social interactions, and confirm emotion identification in social contexts

The Glass provides the selected feedback mechanism whenever it recognizes one of the eight emotions. Families are provided a list of suggested activities divided into three subcategories (“Play”, “Tell me…”, “Others”). Their choice has no impact on the way the system works during the activity, but is meant to provide suggestions to families on which activities to do with their child during a free play session. Once the game starts, the child receives the selected social cue whenever an emotion is detected.

#### Capture the Smile: A semi-structured activity developed to focus on contextual elements of emotions, increase eye contact, and increase social overtures

“Capture the Smile” is a scavenger hunt game for the child, adapted from Picard et al.^71^ The child is prompted by audio cues from the Glass to provoke a specific emotion in another person. The audio prompt, “Find a/an <emotion> face”, uses Glass’ bone-conducting speakers. The child may say the selected emotion, act it out, or provide contextual clues around the emotion in hopes of provoking the other person to express the emotion they are seeking. In this way, the game can be adapted towards both low-functioning and high-functioning children with autism. The app will only provide the audiovisual feedback to the child-wearer, when the person interacting with the child expresses the selected emotion. This game lasts for 3 min, or until the child has successfully evoked 20 emotions, whichever comes first.

#### Guess the Emotion: A structured activity developed to increase eye contact and emotion identification

Guess the Emotion does not use the emotion classifier software. It is entirely controlled by the app. It challenges the child to guess the emotion expressed on the face of the person they interact with. In each round, the parent chooses one of the eight emotions to emote and then waits for the child to make eye contact and guess which of the emotions are being expressed. The parent records the child’s response and if the child guesses the correct emotion, the Glass provides the audiovisual cue corresponding to that emotion. The game is not timed but scores are recorded and reported for the family to track change from one session to the next.

### Evaluations and in-lab procedures

Each participating family was given the Superpower Glass system after completing an onboarding process and assessed for changes in behavioral measures at the end of the treatment period. Participants and the participants’ nuclear family members attended in-person appointments at Stanford University, including an intake appointment, check-in appointments, and a conclusion appointment.

During the intake appointment, the research team collected informed consent from participants, including any family members who may interact with the Superpower Glass device. Parents completed several behavioral evaluations^72–77^ (Table 6). In parallel, the participating child worked with a trained clinical researcher to complete the Nonverbal-Fluid Reasoning and the Verbal-Knowledge sections of the Abbreviated Binet Intelligence Quotient (ABIQ)^76^ to assess IQ. The researcher, who was not blind to the study’s hypothesis, also assessed the child’s facial affect recognition using an in vivo facial affect recognition task (Emotion Guessing Game, “EGG”), a secondary outcome measure developed for the purposes of this study, described in further detail in section “Outcome measures”.

**Table 6:** Evaluations administered to families during appointments

Instrument name	Behaviors measured
Social Communication Questionnaire (SCQ^73,57^)	Measures general ASD symptomatology using social and emotional behaviors.
Social Responsiveness Scale 2 (SRS-2^74^)	Presence and severity of social impairment.
Child Behavior Checklist (CBCL^72^)	Behavioral and emotional problems.
Multidimensional Social Competence Scale (MSCS^77^)	Social competence measurement.
Vineland Adaptive Behavior Scales, 2nd edition (Vineland-II^75^)	Adaptive behavior in communication, daily living skills, socialization, and motor skills.
Stanford Binet Intelligence Scale (ABIQ^76^)	Uses two subdomains of verbal/nonverbal sections to determine IQ.
Emotion Guessing Game (EGG)	A 40-question measure developed to assess how well participants can label the eight emotions examined on a live clinical researcher.

All families received the Superpower Glass system (Google Glass, Android Phone with app, cases, and chargers) to use for 2 months upon completion of their intake questionnaires. Each participating family was asked to complete at least three 20-minute sessions per week during the treatment period, and was encouraged to use the system at will beyond this structured set of sessions. We requested that families complete no more than two sessions per day. Families attended check-in appointments at Stanford University during the treatment period, as the family’s schedule permitted. During each check-in, the study manager administered a semi-structured interview, as well as the EGG evaluation to track participants’ progress with identifying emotions. Data were then pulled from the family’s Superpower Glass system and stored on secure and encrypted cloud infrastructure for the study team to review.

At the conclusion appointment, the Superpower Glass system was returned to the study team and parents completed a phenotype evaluation, described in section “Outcome measures”, while child participants completed the EGG. Additionally, we conducted a semi-structured interview (Table 2) to derive qualitative feedback on the software, hardware, and experience. All user interaction data was pulled and logged from the returned device.

### Outcome measures

To assess the efficacy of our system on its ability to improve social skills for children with ASD, parents completed the Social Responsiveness Scale (SRS-2^74^) at intake and conclusion. The SRS-2 is a peer-reviewed standardized measure to examine ASD severity by social interactions. *T*-Scores below 60 indicate a “Normal” range, scores between 60 and 65 indicate a “mild ASD” range, scores between 65 and 75 indicate a “moderate ASD” range, and scores above 75 indicate a “severe ASD” range. We evaluated the total SRS-2 *t*-scores via repeated measures one-way ANOVA. We also used nonparametric measures on item level SRS-2 data to determine more granular changes that contributed to the total SRS-2 changes. We evaluated the item level changes with a Wilcoxon Rank Sum test as way to examine the feature-level contribution to overall change in the SRS-2 scores, as each question’s response options were ordinal (1 = not true, 2 = sometimes true, 3 = often true, and 4 = almost always true).

To assess the efficacy of our system on its ability to improve children’s facial affect recognition skills, we used a repeated measures one-way ANOVA analysis to analyze EGG scores at intake and at conclusion. We designed EGG to evaluate the child’s ability to correctly label emotions expressed by an examiner, who has been reliably trained to facially express each emotion in real time. EGG is a pre-set list of eight emotions, listed five times each (Happy, Sad, Angry, Afraid, Surprised, Calm, Disgust, and “Meh”/contempt). We used a random order generator to set the order of the 40-question list, which is then used for all EGG evaluations. During this quick 40-question evaluation, the examiner first lists the various emotion choices to the child before beginning the evaluation. Then, the examiner acts out the expressions, and waits for a guess from the child, who labels the emotion. The child guesses which emotion the examiner is expressing and then the examiner records the response and proceeds to the next emotion. The participant does not receive any feedback during or after evaluation.

Lastly, to examine how (a) ASD functioning level (evaluated via ABIQ scores), (b) number of therapies enrolled, and (c) days of usage at home were related to improvements on outcome measures, we ran Pearson’s correlation tests to determine the relationships between each of these independent variables and points of improvement on (a) SRS total *T*-scores from intake to conclusion, and (b) points of improvement on EGG scores from intake to conclusion.

### Data availability

De-identified supporting data, materials, and associated protocols from this manuscript may be made available from the corresponding author upon reasonable request.

### Code availability statement

Code is available from the corresponding author upon reasonable request.

## Electronic supplementary material

Appendix A

## Electronic supplementary material

**Supplementary information** accompanies the paper on the *npj Digital Medicine* website (10.1038/s41746-018-0035-3).
