# Supplementary material for: Exploratory study examining the at-home feasibility of a wearable tool for social-affective learning in children with autism
Source: NPJ Digit Med. 2018 Aug 2;1:32. doi: 10.1038/s41746-018-0035-3 (PMC6550272; doi:10.1038/s41746-018-0035-3)
Supplement: Supplementary file 1 — Appendix A [file 41746_2018_35_MOESM1_ESM.docx]

Appendix A

*“We already noticed something very dramatically I like to share. [Participant 1] is actually looking at us when he talks through google glasses during a conversation and it was noticed without glasses from his teacher in Language Art yesterday, its ^62^ almost like a switch was turned. We found this very important to share and I hope it will help your research to take a closer look at this event when other kids will start wearing the glasses. Thank you!!! My son is looking into my face.” - Participant 1 Parent*

*“I was at [Participant 17]’s school speaking with the office secretary there about something, and in reminiscing about how long [Participant 17] has been at the school she mentioned how [Participant 17] now looks her in the face when speaking to her (whereas previously [Participant 17] wouldn’t look at her). I am seeing improved eye contact at home as well, though some days it’s still hit or miss.” - Participant 17 Parent*

*“It’s helped me to understand some people’s emotions...I can tell when a friend is upset better now than I could before.” “I’ve been applying what I saw with the Google Glass to situations without the Google Glass...I would see my friend’s face and it would look similar to one of the faces I saw on my parents when they were upset so then I could ask my friend, ‘What happened?’” - Participant 3*

*“I have noticed a big difference with her ability to recognize emotions.” - Participant 18 Parent*
